# Expressing accessory proteins in cellulolytic *Yarrowia lipolytica* to improve the conversion yield of recalcitrant cellulose

**DOI:** 10.1186/s13068-017-0990-y

**Published:** 2017-12-11

**Authors:** Zhong-peng Guo, Sophie Duquesne, Sophie Bozonnet, Jean-Marc Nicaud, Alain Marty, Michael Joseph O’Donohue

**Affiliations:** 1 LISBP, Université de Toulouse, CNRS, INRA, INSA, Toulouse, France; 2 Micalis Institute, INRA, AgroParisTech, Université Paris-Saclay, Jouy-en-Josas, France; 3 LISBP-Biocatalysis Group, INSA/INRA UMR 792, 135, Avenue de Rangueil, 31077 Toulouse, France

**Keywords:** *Yarrowia lipolytica*, Cellulolytic biocatalyst, Consolidated bioprocessing, Accessory proteins, Xylanase, Lytic polysaccharide monooxygenase, Swollenin

## Abstract

**Background:**

A recently constructed cellulolytic *Yarrowia lipolytica* is able to grow efficiently on an industrial organosolv cellulose pulp, but shows limited ability to degrade crystalline cellulose. In this work, we have further engineered this strain, adding accessory proteins xylanase II (XYNII), lytic polysaccharide monooxygenase (LPMO), and swollenin (SWO) from *Trichoderma reesei* in order to enhance the degradation of recalcitrant substrate.

**Results:**

The production of EG I was enhanced using a promoter engineering strategy. This provided a new cellulolytic *Y. lipolytica* strain, which compared to the parent strain, exhibited higher hydrolytic activity on different cellulosic substrates. Furthermore, three accessory proteins, *Tr*XYNII, *Tr*LPMOA and *Tr*SWO, were individually expressed in cellulolytic and non-cellulolytic *Y. lipolytica*. The amount of rh*Tr*XYNII and rh*Tr*LPMOA secreted by non-cellulolytic *Y. lipolytica* in YTD medium during batch cultivation in flasks was approximately 62 and 52 mg/L, respectively. The purified rh*Tr*XYNII showed a specific activity of 532 U/mg-protein on beechwood xylan, while rh*Tr*LPMOA exhibited a specific activity of 14.4 U/g-protein when using the Amplex Red/horseradish peroxidase assay. Characterization of rh*Tr*LPMOA revealed that this protein displays broad specificity against β-(1,4)-linked glucans, but is inactive on xylan. Further studies showed that the presence of *Tr*LPMOA synergistically enhanced enzymatic hydrolysis of cellulose by cellulases, while *Tr*SWO1 boosted cellulose hydrolysis only when it was applied before the action of cellulases. The presence of r*Tr*XYNII enhanced enzymatic hydrolysis of an industrial cellulose pulp and of wheat straw. Co-expressing *Tr*XYNII and *Tr*LPMOA in cellulolytic *Y. lipolytica* with enhanced EG I production procured a novel engineered *Y. lipolytica* strain that displayed enhanced ability to degrade both amorphous (CIMV-cellulose) and recalcitrant crystalline cellulose in complex biomass (wheat straw) by 16 and 90%, respectively.

**Conclusions:**

This study has provided a potent cellulose-degrading *Y. lipolytica* strain that co-expresses a core set of cellulolytic enzymes and some accessory proteins. Results reveal that the tuning of cellulase production and the production of accessory proteins leads to optimized performance. Accordingly, the beneficial effect of accessory proteins for cellulase-mediated degradation of cellulose is underlined, especially when crystalline cellulose and complex biomass are used as substrates. Findings specifically underline the benefits and specific properties of swollenin. Although in our study swollenin clearly promoted cellulase action, its use requires process redesign to accommodate its specific mode of action.

**Electronic supplementary material:**

The online version of this article (10.1186/s13068-017-0990-y) contains supplementary material, which is available to authorized users.

## Background

The use of lignocellulosic biomass (LCB) as a manufacturing raw material is regarded as a key feature of the bioeconomy, because it will allow industry to transit to a sustainable, low carbon future [1, 2]. However, processing lignocellulosic biomass in an economically viable way remains a challenge, despite the considerable worldwide efforts.

Cellulose, the main component of lignocellulosic biomass, is built from linear β-glucan chains containing several hundreds of β-1,4-linked glucosyl units. In nature, cellulose exists in both ordered crystalline forms, in which multiple polysaccharide chains are densely packed into microfibrils, and to a lesser extent in disordered amorphous forms. One of the aims of biomass transformation processes is to increase the proportion of amorphous cellulose, since this form is then more amenable for further processing [3]. However, when processing plant cell walls, cellulose crystallinity is not the only hurdle to overcome, since rather like reinforced concrete, cellulose ‘rods’ (bundles of microfibrils) are embedded in a matrix, which in this case is composed of hemicellulose and lignin [4]. Together, these features render lignocellulosic biomass particularly recalcitrant to all but the most severe processing strategies [5].

Biochemical conversion of lignocellulose into target products usually occurs in three macro-operations. Biomass pretreatment often involves a combination of physical and chemical strategies that are deployed to modify the structure of lignocellulosic biomass, separating cellulose from lignin and hemicellulose, reducing complexity and increasing the accessible surface area [6]. Pretreatment is followed by enzymatic depolymerization of the polysaccharides, generating fermentable sugars using cellulases and hemicellulases. Finally, fermentation using a suitable microorganism is employed to transform sugars into the desired product(s) [7]. Among these steps, pretreatment and enzymatic hydrolysis are still significant cost drivers, with enzyme loadings and thus costs still exceeding those that have been targeted for many years [8].

One way to reduce cost in biomass processing is to integrate some of the steps and reduce or eliminate the need for external commercial enzymes. This is known as consolidated bioprocessing (CBP) and to achieve this, it is necessary to employ a cellulolytic microorganism (or a microbial consortium) that can both hydrolyse biomass polysaccharides and convert sugars into a target product [9]. However, despite the attractiveness of this concept, it is difficult to achieve because very few naturally occurring microorganisms can perform these two functions in an economically viable and industrially compatible manner [10]. Therefore, it is pertinent to look towards microbial strain engineering strategies in order to confer to an engineered microorganism the attributes required for CBP.

To engineer a microorganism for CBP purposes, it is most common to first select a microorganism that displays a suitable metabolism for target product manufacture and then confer it with cellulolytic capability, using heterologous expression of cellulases of fungal or bacterial origin [9]. Using this approach, several engineered CBP microorganisms have been successfully produced (reviewed in [9, 11]). However, these have mostly been tested using model cellulose substrates that are excessively amenable to enzyme hydrolysis and/or produce low product titers [7]. As a result, when such engineered CBP strains are confronted with industrial cellulose pulps, the addition of exogenous enzymes is necessary to achieve complete hydrolysis [11].

The extensively studied cellulolytic secretome of the soft-rot fungus *T. reesei* (syn. *Hypocrea jecorina*) contains several cellulases that synergistically act on complex substrates [12]. In the *T. reesei* cellulolytic secretome, EG I and EG II are the main endo-acting enzymes, representing approximately 15 and 10% (w/w) of the total protein content, respectively [13], while CBH I and CBH II (Cel7A and Cel6A) are the major exo-acting components, representing 50 and 20% of the total protein content, respectively [1]. The minimal requirement for a cellulase cocktail composed of free enzymes includes at least one endoglucanase (EG, EC 3.2.1.4), one cellobiohydrolase (CBH, EC 3.2.1.91) and a β-glucosidase (BGL, EC 3.2.1.21) [14], the latter being necessary to ensure the production of glucose from cellodextrins. Nevertheless, most commercial cocktails are more complex and are completed with a range of accessory enzymes, including hemicellulases, cinnamic acid and acetyl esterases that together remove hemicellulose and thus increase cellulose accessibility and decrease the risk of cellulase inhibition by xylo-oligosaccharides [15, 16]. Until recently, assembling a sufficiently comprehensive array of polysaccharide hydrolases in a cocktail constituted the main strategy to achieve the hydrolysis of cellulose pulps. However, the recent discovery of lytic polysaccharide monooxygenases (LPMOs) has dramatically changed this view and opened up new options for the engineering of efficient cellulolytic microbial cell factories [8].

Belonging to the so-called Auxiliary Activity family 9 (AA9, formerly GH61) of the CAZy classification [17], LPMOs have been shown to significantly boost the overall efficiency of cellulose hydrolysis when using canonical hydrolytic cellulases. Studies reveal that the LPMOs catalyze the oxidative cleavage of insoluble polysaccharides using molecular oxygen or peroxide and an electron donor [18, 19]. Although most characterized LPMOs oxidize polysaccharides at the C1 position (type 1 PMOs), yielding lactone [19–21], some perform oxidation at the C4 position (type 2 PMOs) or at both the C1 and C4 positions (type 3 PMOs), or at C6. In all cases, the action of LPMOs leads to the formation of a ketoaldose [20, 22, 23]. Considering the clear benefits of LPMOs for cellulase-mediated hydrolysis of cellulose, enzyme manufacturers have incorporated these into the latest generation of commercial cellulase cocktails [24].

In addition to LPMOs, it has been known for some time that fungal cellulolytic secretomes and plant cell walls contain non-enzymatic proteins that possess the ability to disrupt the ordered hydrogen-bonding network in crystalline cellulose. Although the exact role of proteins such as swollenins (fungal origin) and expansins (plant origin) has not been fully elucidated, research reveals that they enhance cellulose-mediated hydrolysis of cellulose, probably by disrupting the cellulose surface, making it more amenable to attack by cellulases [25–27].

Recently, we conferred cellulolytic properties to *Yarrowia lipolytica*, an oleaginous yeast that is recognized for its industrial usefulness and safety (it is classified by the FDA as a Generally Recognized As Safe strain) [28, 29]. To achieve this, we expressed in a *Y. lipolytica* strain a range of enzymes including BGLs, EGs, and CBHs, taking care to control the relative proportions of each of these in order to obtain a combination that is quantitatively similar to that of the native cellulase system of *T. reesei*. The engineered cellulolytic *Y. lipolytica* was shown to grow efficiently on industrial cellulose pulp (CIMV-cellulose, mostly amorphous), but displayed some difficulty to degrade recalcitrant crystalline cellulose [30]. Therefore, in the present work, we describe how the cellulolytic *Y. lipolytica* strain has been further manipulated to increase the hydrolysis of crystalline cellulose and complex substrates. This has been achieved by altering the proportions of the expressed cellulases and adding accessory proteins XYNII, LPMO, and swollenin.

## Results and discussion

### Enhancing the production of *T. reesei* endoglucanase I in cellulolytic *Y. lipolytica*

**Fig. 1:**
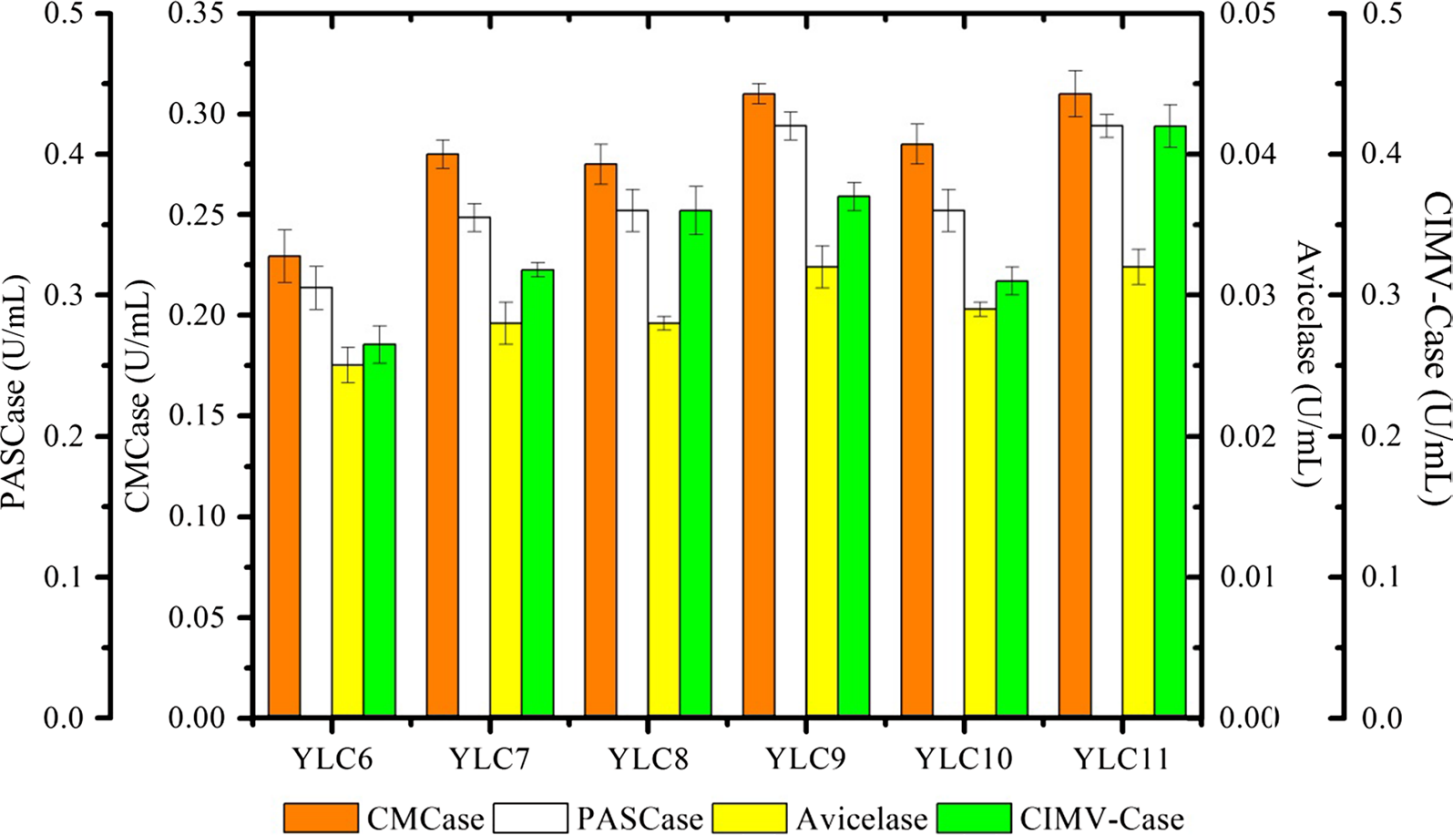
Comparison of the hydrolytic activity (mg reducing sugar/min/mL supernatant) on various cellulosic substrates of the secretomes produced by cellulolytic *Y. lipolytica* strains YLC6 (∆poxB12 strain expressing EG I, EG II, CBH I, and CBH II), YLC7 (YLC6 + enhanced EG I expression), YLC8 (YLC7 + XYN II), YLC9 (YLC7 + LPMOA), YLC10 (YLC7 + SWO1), and YLC11 (YLC7 + XYNII + LPMOA)

Previous studies have revealed that the efficient hydrolysis of pretreated LCB requires the presence of 25–35% (w/w) EG I in the cellulase cocktail, and that EG I cannot be replaced by EG II [31–33]. Additionally, our results showed that recombinant EG I exhibits a twofold higher specific activity on insoluble substrates, such as Avicel, β-1,3 and β-1,4 glucans, than EG II. Together, these findings underline the important role of EG I for the hydrolysis of recalcitrant biomass. Unfortunately, in the previously described engineered cellulolytic *Y. lipolytica* strain (YLC6), the secretion yield of *T. reesei* EG I was approximately 60% lower than that of EG II [30]. Therefore, to alter this ratio, a hybrid promoter strategy [34] was employed, modifying the core TEF promoter element controlling EG I expression. Following successful promoter modification, analyses revealed that all of the previously introduced genes were present (Additional file 1: Figure S1) and that the new strain, YLC7, was able to hydrolyze CMC, PASC, and Avicel cellulose (Fig. 1). Comparing the performance of YLC7 to the parental strain YLC6 revealed that HTEF promoter-controlled expression of r*Tr*EG I led to 18, 14, 10, and 17% increases in hydrolytic activity on CMC, PASC, Avicel, and CIMV-Cellulose, respectively. Using anti-His Western blot analysis, it was possible to correlate these increases with the improved expression level of rh*Tr*EG I in the secretome of YLC6 (Additional file 1: Figure S2). Since our previous results showed that His6-tagging did not influence r*Tr*EG I production [30], it is reasonable to attribute the increase in hydrolytic activity of YLC7 to its higher r*Tr*EG I expression, although the reported cellulolytic activity is the composite result of all secreted cellulase activity.

### Expression and characterization of *T. reesei* xylanase II in *Y. lipolytica*

**Fig. 2:**
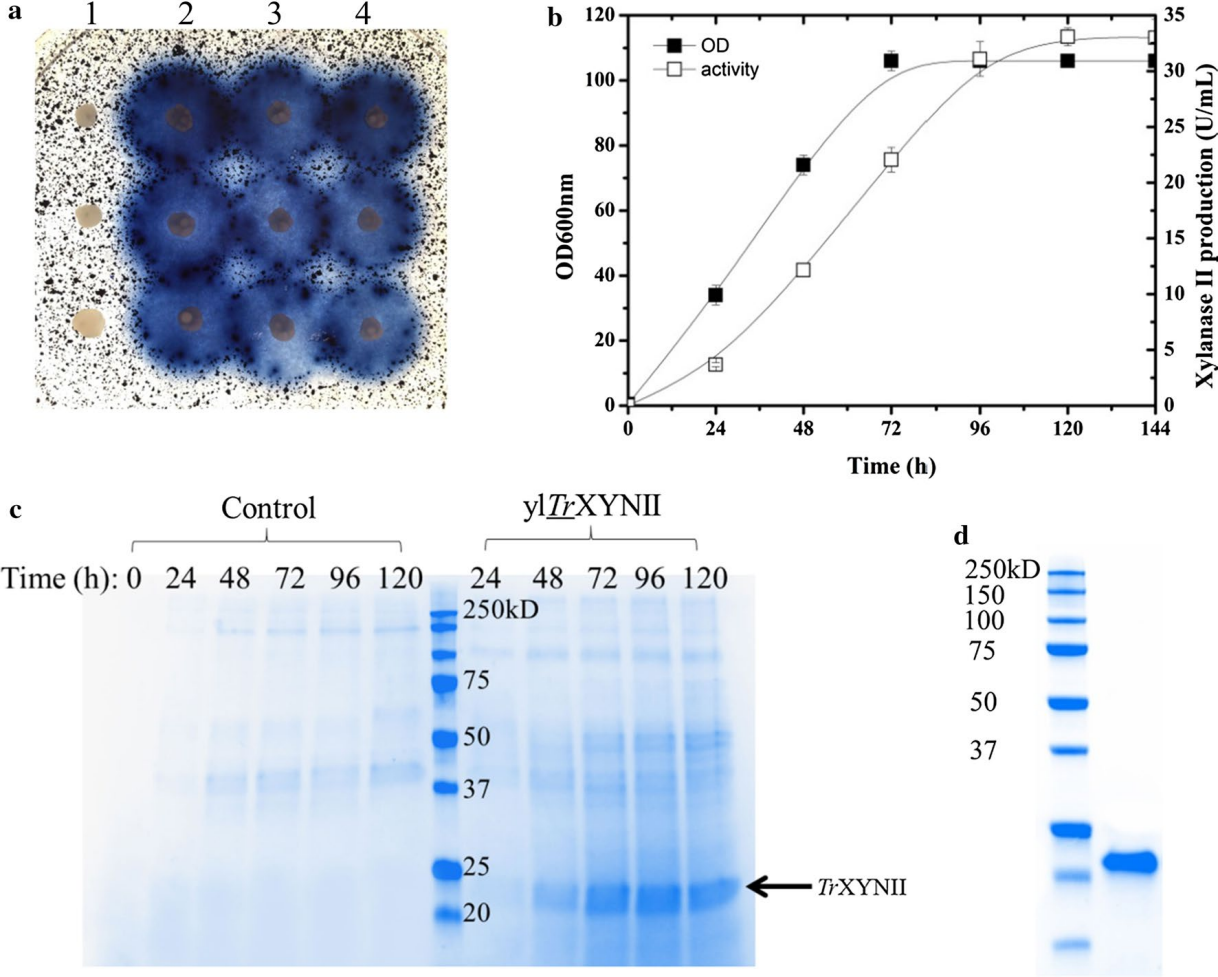
Expression of r*Tr*XYNII in *Y. lipolytica*. **a** Screening of *Y. lipolytica* transformants on agar plates containing 0.2% w/v AZCL-arabinoxylan: lane 1, *Y. lipolytica*-control; lane 2 to 3, *Y. lipolytica* expressing r*Tr*XYNII and rh*Tr*XYNII respectively; L4, YLC8; **b** r*Tr*XYNII production on YTD medium versus time; **c** SDS-PAGE analysis of the culture supernatant of *Y. lipolytica* control strain expressing empty plasmid and the recombinant strain expressing rh*Tr*XYNII; **d** SDS-PAGE analysis of the rh*Tr*XYNII after purification, protein marker (lane 1) and rh*Tr*XYNII (lane 2)

The results of previous studies focused on the design of optimal cellulase formulations for complex biomass hydrolysis have emphasized the requirement for xylanase, which acts synergistically with cellulases [31–33]. Accordingly, we expressed the *T. reesei* xylanase II (r*Tr*XYNII) in *Y. lipolytica* JMY1212 under TEF promoter control and using the lipase 2 pre-pro region to facilitate protein secretion, characterized the resultant recombinant enzyme, and then investigated how r*Tr*XYNII could be expressed in the genetic background of YLC7. Screening on solid medium containing AZCL-arabinoxylan of *Y. lipolytica* transformants readily revealed the presence of clones producing either r*Tr*XYNII or its His-tagged variant rh*Tr*XYNII (Fig. 2a). To further confirm successful expression of *Tr*XYNII, positive clones were grown in liquid YTD medium and xylanase activity was assayed in the culture supernatant. Accordingly, it was possible to demonstrate that during cultivation xylanase activity steadily increased over a 96-h period, reaching a final activity (in the case of rh*Tr*XYNII) of 32.0 U/mL (Fig. 2b). Furthermore, using SDS-PAGE the comparison of the rh*Tr*XYNII-containing culture supernatant to that of a control culture (Fig. 2c) revealed the presence of a discrete species migrating to a position corresponding to an approximate Mw of 22 kDa, which is consistent with the theoretical Mw of *Tr*XYNII (22.0 kDa).

In order to further investigate the properties of rh*Tr*XYNII, the protein was purified from the culture supernatant (Fig. 2d) and its activity was measured on beechwood xylan in different conditions of pH and temperature. This revealed that rh*Tr*XYNII reached optimal activity (532 U/mg-protein) at pH 6.0 and 60 °C (Additional file 1: Figure S3), and that higher temperatures led to rapid enzyme inactivation (data not shown). These findings are consistent with previous data obtained for r*Tr*XYNII expressed in *Pichia pastoris* [35]. Knowing the specific activity of rh*Tr*XYNII, it was also possible to calculate that 62 mg/L of rh*Tr*XYNII was secreted by *Y. lipolytica*, when growing in batch mode in YTD medium contained in flasks.

### Expression of *T. reesei* lytic polysaccharide monooxygenase A in *Y. lipolytica*

**Fig. 3:**
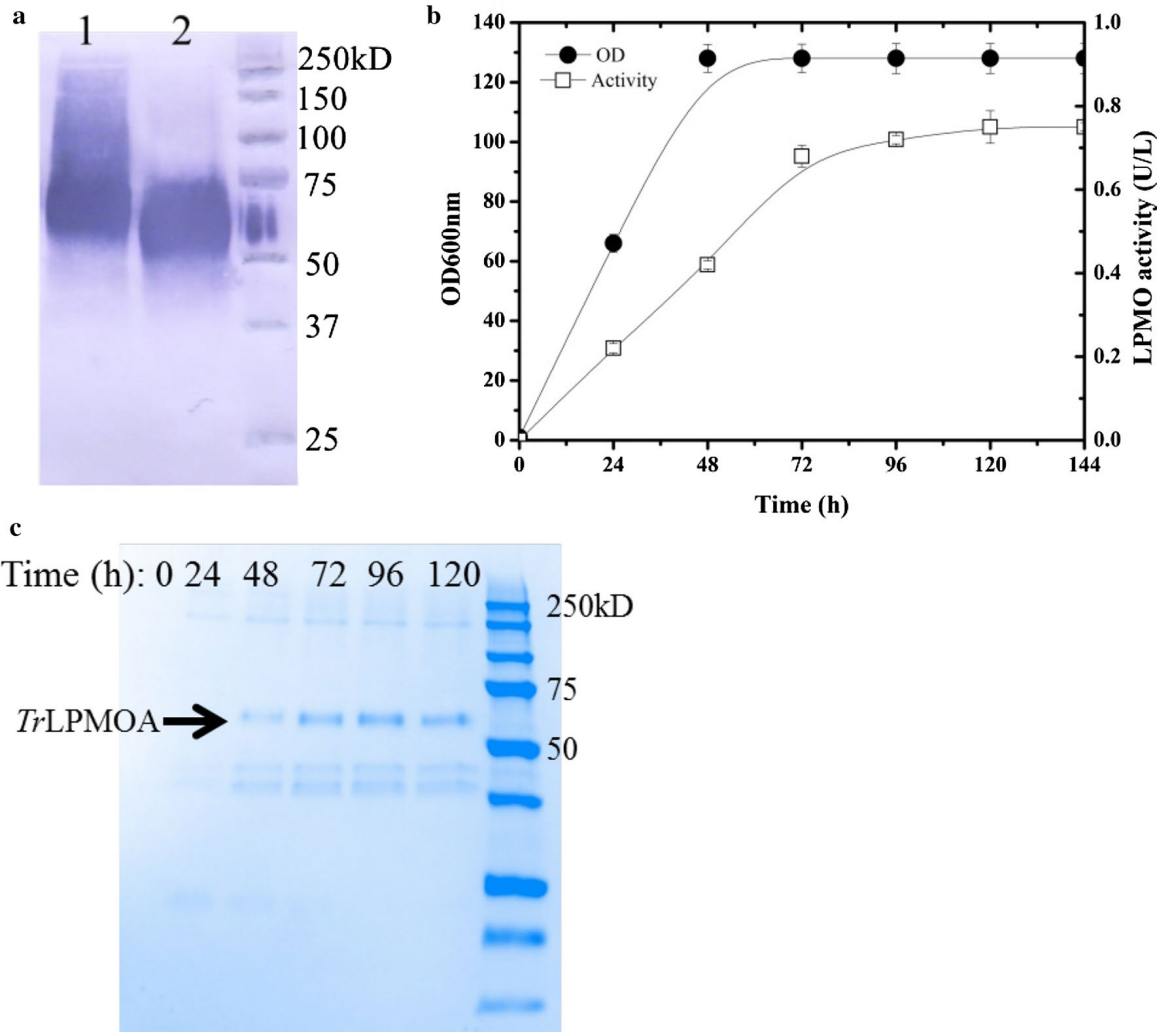
Expression of *Tr*LPMOA in *Y. lipolytica*. **a** Western blot analysis of the heterologous LPMOA protein secreted by *Y. lipolytica*: lane 1, rh*Tr*LPMOA; lane 2, Endo H-treated rh*Tr*LPMOA; **b** time-related enzyme production by yeast growing in liquid YTD; **c** SDS-PAGE analysis of the culture supernatant of *Y. lipolytica* transformants

In the *T. reesei* secretome LPMOs form a very minor component [36]. However, recent in vitro studies have shown that the inclusion of these enzymes in cellulase cocktails can reduce overall enzyme loadings, while maintaining the efficiency of cellulose conversion [37, 38]. Therefore, considering that it would be useful to include *Tr*LPMOA in the cellulase cocktail expressed by YLC7, we cloned and expressed a His-tagged rh*Tr*LPMOA in *Y. lipolytica* JMY1212 under TEF promoter control and using the lipase 2 pre-pro region to facilitate protein secretion. Successful secretion of rh*Tr*LPMOA into the culture supernatant was first established by SDS-PAGE and anti-His Western blot analyses. These revealed a smear in the Mw range 60–200 kDa, suggesting that the expected 34.4 kDa protein species was glycosylated (Fig. 3), which is unsurprising since yeasts are well known to perform hyper *N*-mannosylation [39]. To investigate this, rh*Tr*LPMOA was submitted to Endo H-mediated deglycosylation and further SDS-PAGE/Western blot analysis, which revealed a better defined protein population that migrated to a position correlating to a median Mw of 55 kDa (Fig. 3a). This result suggests that even after the removal of N-glycosyl moieties, rh*Tr*LPMOA still bears additional post-translational modifications, possibly *O*-glycosylation of serine/threonine-rich linker region [40].

To confirm that the expressed recombinant protein was active, the ability of rh*Tr*LPMOA to mediate the reduction of O_2_ to H_2_O_2_ in the presence of ascorbate was established. Moreover, using this test, it was shown that the expression level of rh*Tr*LPMOA steadily increased over a 72-h period, reaching a final activity in the culture supernatant of 0.75 U/L (Fig. 3b).

### Characterization of the rh*Tr*LPMOA expressed in *Y. lipolytica*

**Fig. 4:**
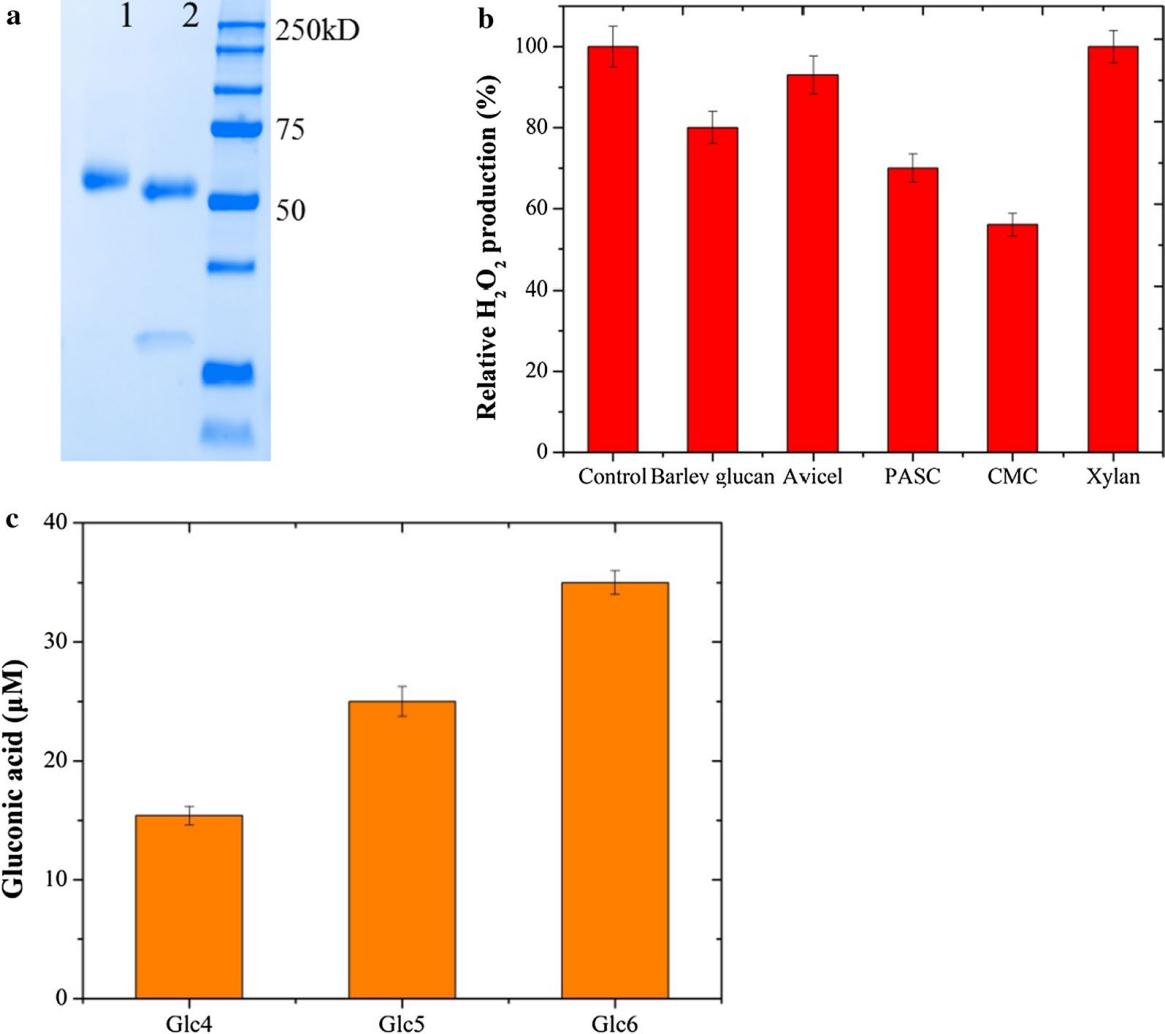
Characterization of *Tr*LPMOA expressed in *Y. lipolytica*. **a** SDS-PAGE analysis of the purified rh*Tr*LPMOA (lane 1) and rh*Tr*LPMOA treated by Endo H (lane 2); **b** H_2_O_2_ generation by rh*Tr*LPMOA in the presence and absence of various oligosaccharides substrates; **c** gluconic acid produced from cello-oligosaccharides (DP4-DP6) by action of rh*Tr*LPMOA in the presence of ascorbic acid

To further assess the functionality of rh*Tr*LPMOA, the protein was purified in a single step, with an overall yield > 60% (Fig. 4a) and characterized. The purified rh*Tr*LPMOA exhibited a specific activity of 14.4 U/g. This value is similar to that of the *Neurospora crassa* LPMO9F (15 U/g) expressed in *P. pastoris*, which is the most active LPMO reported so far using the same activity assay [8]. The amount of rh*Tr*LPMOA secreted by *Y. lipolytica* in YTD medium during batch cultivation in flasks was approximately 52 mg/L, a yield that is moderate compared to other examples of LPMO heterologous expression [8, 41].

To further investigate the substrate specificities of rh*Tr*LPMOA, we measured the production of H_2_O_2_ in the presence of 1.0% (w/v) PASC, CMC, Avicel, barley glucan (β-1,3; 1,4), and beechwood xylan. The repression in H_2_O_2_ production was observed for rh*Tr*LPMOA in the presence of the four glucose-polymers (Fig. 4b) while xylan had no effect on H_2_O_2_ production. These results suggest that rh*Tr*LPMOA displays broad specificity on β-(1,4)-linked glucans, but is inactive on xylan.

To further investigate the regioselectivity of rh*Tr*LPMOA, tests were performed using cellodextrins (DP4-6). These revealed the production of d-gluconic acid, confirming that oxidation occurred at C1 (Fig. 4c). Moreover, HPAEC analysis failed to reveal any C4-oxidized species (data not shown). Taken together, these results indicate that rh*Tr*LPMOA displays type 1-like LPMO activity, a conclusion that contradicts previous sequence and phylogeny analyses that suggest it is a type 3 LPMO (i.e., one that oxidizes at both the C1 and C4 positions) [40]. However, these apparently conflictual findings can be resolved by attributing *Tr*LPMOA to a recently described type 3 subgroup (PMO3*) that contains LPMOs such as the one from *Myceliophthora thermophila* AA9 LPMO (MYCTH_92668) that also only performs oxidation at C1 [40].

### Expression and characterization of *T. reesei* swollenin 1 in *Y. lipolytica*

**Fig. 5:**
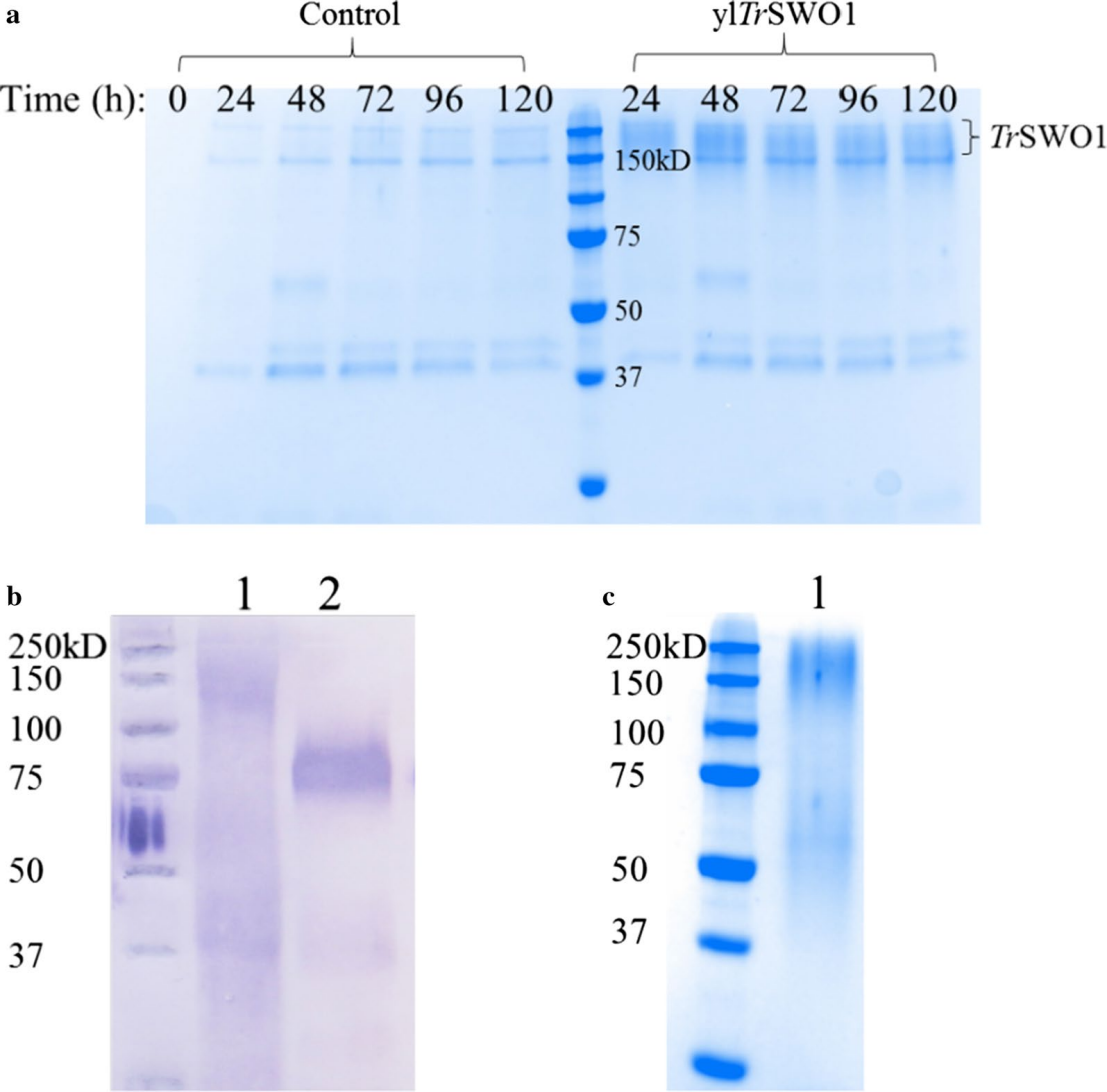
Expression of *Tr*SWO1 in *Y. lipolytica*. **a** SDS-PAGE analysis of the culture supernatant of *Y. lipolytica* control strain expressing empty plasmid and the recombinant strain expressing rh*Tr*SWO1; **b** Western blot analysis of the heterologous SWO1 protein produced by *Y. lipolytica*: lane 1, rh*Tr*SWO1; lane 2, rh*Tr*SWO1 treated by Endo H; **c** SDS-PAGE analysis of the purified rh*Tr*SWO1

In this work, swollenin 1 (SWO1), the major expansin-like protein produced by *T. reesei* [36], was first expressed independently in *Y. lipolytica* JMY1212 under TEF promoter control and using the lipase 2 pre-pro region to facilitate protein secretion. Compared to a control culture, SDS-PAGE analysis of culture supernatants of *Y. lipolytica* transformants expressing rh*Tr*SWO1 revealed the presence of a smear (150–200 kDa), which is much higher than the expected Mw of rh*Tr*SWO1 (50 kDa) (Fig. 5a). However, Western blot analysis using anti-His antibody confirmed that this large protein species was His-tagged (Fig. 5b), indicating that it is a highly glycosylated variant of rh*Tr*SWO1, a finding that concurs with data in the literature [26, 27]. Using NetNGlyc 1.0 (http://www.cbs.dtu.dk/services/NetNGlyc/) to analyze the amino acid sequence of rh*Tr*SWO1 has predicted that SWO1 bears three potential *N*-glycosylation sites. Therefore, to explore this possibility the protein was treated with Endo H and analyzed by Western blot. This revealed a newly formed discrete protein species that migrates to position corresponding to a Mw of approximately 75 kDa (Fig. 5b), a result consistent with the analysis of native SWO1 isolated from the *T. reesei* secretome [27]. Presumably the discrepancy between the predicted Mw for the polypeptide sequence of SWO1 and that measured (75 KDa) is due to other post-translational modifications, such as *O*-glycosylation of serine/threonine-rich linker region [26]. Finally, to further investigate the functionality of rh*Tr*SWO1, the protein was purified (Fig. 5c).

### Combining the action of LPMOA or SWO1 with cellulases for Avicel hydrolysis

**Fig. 6:**
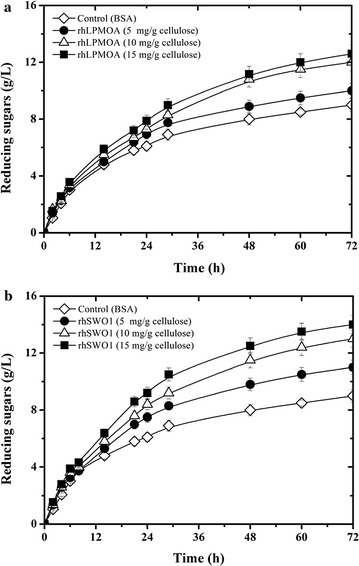
Hydrolysis of Avicel (**a**) in the presence of rhLPMOA at different concentrations; **b** after pretreatment with swollenin at different concentrations

To investigate the synergy of rh*Tr*LPMOA and rh*Tr*SWO1 with cellulases, tests were performed on microcrystalline cellulose (Avicel) using a commercial cellulase cocktail (Celluclast) supplemented with each recombinant protein at different concentrations. Celluclast was used because, unlike Cellic™ CTEC2 [37], it is devoid of LPMOs and SWO. Adding rh*Tr*LPMOA alone significantly accelerated cellulose hydrolysis (Fig. 6a), with rate being dependant on the concentration of rh*Tr*LPMOA. When using 15 mg rh*Tr*LPMOA per g cellulose, the conversion of Avicel (20 g/L) to soluble reducing sugars reached 60% within 72 h, which constitutes a 40% increase compared with the control (Fig. 6a). In contrast, adding rh*Tr*SWO1 alone failed to produce any measurable effect on cellulose hydrolysis, a result that is consistent with previous data and suggests that swollenin does not display synergy with EG and CBH [42]. Nevertheless, the incubation of Avicel with rh*Tr*SWO1 (5–15 mg rh*Tr*SWO1/g cellulose) for a 24-h period prior to the reaction enhanced Avicel hydrolysis, with up to 70% conversion to reducing sugars occurring after 72 h when using 15 mg rh*Tr*SWO1/g cellulose (Fig. 6b), although higher loadings did not procure further improvements (data not shown). Overall, these data indicate that swollenin adsorption is a prerequisite for amorphogenesis and that this is governed by a time-dependant equilibrium. For optimal Avicel amorphogenesis, it is necessary to leave sufficient time for several cycles of adsorption/desorption to occur [26, 42]. Finally, it is noteworthy that when using a similar protein load, the effect of rh*Tr*SWO1 on cellulase-mediated Avicel hydrolysis was more potent than that of rh*Tr*LPMOA, indicating that the use of SWO instead of LPMO in a biorefinery process might be more cost-effective. However, for this to be feasible, it would be necessary to propose a redesigned process and account for the cost burden associated with the time needed for the action of SWO.

### Construction of recombinant *Y. lipolytica* strains expressing multiple cellulases and accessory enzymes

**Fig. 7:**
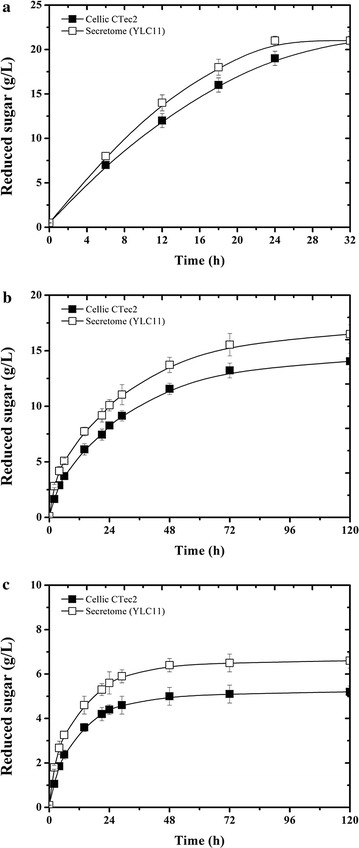
Comparison of hydrolytic efficiency of the secretome of YLC11 and Cellic CTec2 in enzymatic hydrolysis of **a** CIMV-cellulose, **b** Avicel, and **c** wheat straw at the enzyme loading of 10 FPU/g-cellulose

To further enhance the potency of cellulose hydrolysis by an engineered *Y. lipolytica*, the genes encoding *Tr*XYNII, *Tr*LPMOA, and *Tr*SWO1 were singly expressed in the cellulase-producing strain YLC7 (Table 1). The presence of all of the exogenous genes was established using PCR (Additional file 1: Figure S1). In case of YLC8 (i.e., expressing r*Tr*XYNII), subsequent screening on two different solid culture media containing AZCL-arabinoxylan and Azo-CMC respectively, revealed that clones displayed both xylanase and cellulase activity (Additional file 1: Figure S4; Fig. 2). Testing the ability of the supernatants of each of the daughter strains, YLC8 (r*Tr*XYNII), YLC9 (r*Tr*LPMOA), and YLC10 (r*Tr*SWO1), respectively, to hydrolyze CMC, PASC, Avicel, and CIMV-cellulose (containing hemicellulose), revealed contrasting results (Fig. 1). A 12% increase in glucose production compared to YLC7 was procured by YLC8 on CIMV-cellulose hydrolysis, indicating that the presence of a xylanase is specifically beneficial to hydrolyze industrial cellulose pulp. This suggests that the xylanase works synergistically with the cellulases probably by removing hemicelluloses wrapped around glucan microfibrils and thus increasing the cellulose accessibility [43]. Indeed, the action of the xylanase was further confirmed by HPAEC analysis (data not shown), which revealed the presence of xylodextrins among the hydrolysis products. The presence of *Tr*LPMOA (YLC9) enhanced the hydrolysis of all of the substrates tested, which is consistent with its ability to oxidize cellulose (Fig. 1). In contrast, the co-expression of r*Tr*SWO1 with the cellulases (YLC10) did not procure a significant improvement of hydrolysis, consistent with the observation that r*Tr*SWO1 needs sufficient contact time and confirming that the mode of action of this protein is incompatible with a co-expression strategy. For this reason, and in view of the other results, only r*Tr*XNYII and *Tr*LPMOA were retained for co-expression in the genetic background of YLC7, thus producing YLC11. When using the latter strain for hydrolysis tests on the different substrates, it became clear that the highest enhancement was procured on CIMV-cellulose, while the hydrolysis of pure cellulose (CMC and Avicel) did not change when compared to YLC9 (Fig. 1). We further compared the hydrolytic efficiency of the YLC11 secretome with that of the commercial cocktail Cellic CTec2, using CIMV-cellulose, Avicel and wheat straw as substrates. The results showed that when using equivalent enzyme loadings (10 FPU/g-cellulose) the YLC11 secretome degraded all three substrates faster than Cellic CTec2 (Fig. 7). The CIMV-cellulose is the most amenable substrate, since the YLC11 secretome achieved 95% conversion of this substrate to glucose in 24 h, while Cellic CTec2 achieved 86% over the same time period (Fig. 7a). However, the superiority of the YLC11 secretome is better illustrated by its action on Avicel and wheat straw. On these substrates, the secretome achieved 75 and 30% conversion, respectively, whereas the action of Cellic CTec2 yielded 63% conversion of Avicel and 24% conversion of cellulose in wheat straw (Fig. 7b, c). When compared to the commercial cocktail, the higher potency of the YLC11 secretome can probably be attributed to several factors, the first being the presence of a more efficient CBH I [30] Secondly, the ratio of the different enzymes is optimized in the YLC11 secretome and, thirdly, the amount of LPMO present is probably higher. Additionally, the presence of xylanase in the YLC11 secretome is not doubt useful for the hydrolysis of wheat straw. Therefore, we conclude that YLC11 is a particularly relevant strain for use with industrial cellulose pulp and recalcitrance biomass.

**Table 1:** Microbial strains used in the present study

Strains	Relevant genotype	Source of reference
*T. reesei* QM9414	Wildtype	DSMZ
*E. coli* DH5	Φ80dlacZΔm15, *recA1*, *endA1*, *gyrA96*, *thi*-*1*, *hsdR17* (rk^−^, mk^+^), *supE44*, *relA1*, *deoR*, Δ(*lacZY*A-argF) U169	Invitrogen
*Y. lipolytica* JMY1212 (Zeta)	*MATA*, *ura3*-*302*, *leu2*-*270*-*LEU2*-*zeta*, *xpr2*-*322 ∆lip2*, *∆lip7*, *∆lip8*	[52]
yl*Tr*XYNII	Zeta, *pTEF*-*XYNII*-*His6*	This investigation
yl*Tr*LPMOA	Zeta, *pTEF*-*LPMOA*-*His6*	This investigation
yl*Tr*SWO1	Zeta, *pTEF*-*SWO1*-*His6*	This investigation
*Y. lipolytica* YLC6	∆*pox*B12, *pTEF*-*EGI*, *pTEF*-*EGII*; *pHTEF*-*NcCBHI*, *pHTEF*-*TrCBHII*	[30]
YLC6b	∆*pox*B12, *pTEF*-*EGI*-*His6*, *pTEF*-*EGII*; *pHTEF*-*NcCBHI*, *pHTEF*-*TrCBHII*	This investigation
YLC7	∆*pox*B12, *pHTEF*-*EGI*, *pTEF*-*EGII*; *pHTEF*-*NcCBHI*, *pHTEF*-*TrCBHII*	This investigation
YLC7b	∆*pox*B12, *pHTEF*-*EGI*-*His6*, *pTEF*-*EGII*; *pHTEF*-*NcCBHI*, *pHTEF*-*TrCBHII*	This investigation
YLC8	YLC7, *pTEF*-*XYN II*	This investigation
YLC9	YLC7, *pTEF*-*LPMOA*	This investigation
YLC10	YLC7, *pTEF*-*SWO1*	This investigation
YLC11	YLC7, *pTEF*-*XYNII*, *pTEF*-*LPMOA*	This investigation

### The growth of recombinant *Y. lipolytica* strains on cellulosic feedstocks

To investigate the suitability of the different strains described in this study for use in a consolidated bioprocessing concept, each of them (YLC6 to 11) was grown in liquid defined medium containing either CIMV-cellulose, Avicel, or milled wheat straw as the carbon source. Results revealed that all of the strains were able to use CIMV-cellulose, with conversion yields of more than 50% being achieved. In contrast, Avicel and wheat straw were less amenable to hydrolysis (Table 2). In terms of microbial biomass yields, these reached approximately 0.4 g-DCW/g-cellulose on CIMV-cellulose, but only (average) 0.3 and 0.1 g-DCW/g-cellulose on Avicel and wheat straw, respectively. It is noteworthy that the increased expression of r*Tr*EGI (i.e., comparing YLC7 to YLC6) resulted in a slight increase in the conversion of all the cellulosic substrates, and predictably the expression of r*Tr*XYNII (YLC8) enhanced the conversion of CIMV-cellulose (64.0 ± 0.4%), while not affecting Avicel conversion. Interestingly, the expression of r*Tr*XYNII also enhanced the conversion of wheat straw (12.3 ± 0.5%), consistent with the fact that significant amounts of xylan are present in this substrate. Strain YLC9 expressing *Tr*LPMOA consumed 65% of CIMV-cellulose, 36% of Avicel, and 16% of cellulose in wheat straw, results that are comparable or better than those obtained with YLC7 and 8 (producing r*Tr*XYNII). When both r*Tr*XYNII and r*Tr*LPMOA (YLC11) were present, the conversion of CIMV-cellulose and wheat straw were further enhanced (68 and 18%, respectively). Finally, while the treatment with r*Tr*SWO1 (15 mg-swollenin/g-cellulose) before strain cultivation failed to affect CIMV-cellulose conversion, it did prove to be beneficial for Avicel and wheat straw, with YLC11-mediated conversion rates being 42% (Avicel) and 22% (wheat straw), respectively, after 5 days of growth. However, it is also noteworthy that CIMV-cellulose is obtained using organosolv technology, which is known to produce relatively pure cellulose [44]. In further work, it will be interesting to confront YLC11 with cellulose pulps produced using less costly, but more ‘quick and dirty’ technologies [45].

**Table 2:** Comparison of cellulose utilization and biomass yield of cellulolytic *Y. lipolytica* grown on different cellulosic substrates for 120 h in aerobic cultivation

Strains	CIMV-cellulose consumed %	Biomass yield %	Avicel consumed %	Biomass yield %	Cellulose consumed % (wheat straw)	Biomass yield %
YLC6	58.6 ± 0.5	0.41 ± 0.03	30.2 ± 0.3	0.32 ± 0.03	9.7 ± 0.6	0.12 ± 0.01
YLC7	61.5 ± 0.6	0.40 ± 0.05	32.0 ± 0.5	0.31 ± 0.02	10.5 ± 0.7	0.11 ± 0.01
YLC8	64.0 ± 0.4	0.40 ± 0.02	31.8 ± 1.0	0.31 ± 0.02	12.3 ± 0.5	0.12 ± 0.01
YLC9	65.4 ± 0.7	0.39 ± 0.03	36.3 ± 0.9	0.29 ± 0.03	15.8 ± 0.9	0.15 ± 0.01
YLC10	59.0 ± 1.5	0.40 ± 0.04	31.0 ± 1.2	0.31 ± 0.04	10.3 ± 0.9	0.11 ± 0.02
YLC11	67.8 ± 1.0	0.39 ± 0.02	36.5 ± 0.7	0.29 ± 0.02	18.4 ± 0.8	0.15 ± 0.02
YLC11 + S^a^	68.3 ± 1.3	0.38 ± 0.01	42.0 ± 1.1	0.30 ± 0.04	21.6 ± 0.5	0.17 ± 0.01

The results were calculated from at least three biological replicates, and are given as the mean value ± standard deviation. The initial cellulose content was 25 g/L for all the substrates

^a^The cellulose was treated by SWO1 at the dosage of 15 mg/g cellulose for 24 h before enzymatic hydrolysis

Among the different accessory proteins that have been used in this study, LPMOA stands out, because it requires an electron donor and oxygen to function. Therefore, when tests were performed, ascorbic acid was included in the reactions. However, for CIMV-cellulose and wheat straw, this was unnecessary as similar yields of cellulose conversion and biomass production were achieved for YLC9 and YLC11 without the supplementation of ascorbic acid (Additional file 1: Table S2), probably because lignin present in the substrates acts as an electron donor [46]. Additionally, it has been shown that when using conventional cellulase cocktails supplemented with LPMOs, enhancement of cellulose hydrolysis will only occur under aerobic conditions [47]. In this respect, it is noteworthy that *Y. lipolytica* is strictly aerobic, thus the production of LPMOA in CBP-mode should be quite advantageous [29]. Finally, it is also important to note that *Y. lipolytica* can assimilate gluconic acid, an important attribute when LPMOA is present (Additional file 1: Figure S5).

Regarding swollenins, our study clearly confirms the great potential for these proteins, but underlines their incompatibility with a straightforward CBP concept. To draw benefit from swollenins, it will be necessary to devise a hybrid process in which exogenously produced swollenins are used at some point during biomass pretreatment, taking into account their specific operating requirements (i.e., contact time, temperature, and protein loading).

Finally, this study confirmed the usefulness of xylanases in the context of cellulose hydrolysis. Based on our findings and those of previous studies [48], we postulate that further engineering of *Y. lipolytica* to confer additional β-d-xylosidase activity and the ability to metabolize xylose efficiently [49] will procure a quite potent CBP strain. If successful, this could pave the way for the use of *Y. lipolytica* in the biorefinery industry.

## Conclusion

In the present study, a *Y. lipolytica* strain co-expressing core cellulolytic enzymes and accessory proteins has been successfully developed. The results reveal that accessory proteins can significantly enhance cellulose hydrolysis and confer cellulolytic *Y. lipolytica* with the ability on one hand to better handle industrial pulps containing xylan and on the other to hydrolyze more recalcitrant crystalline substrates. Overall, the data presented here confirm the feasibility of a *Y. lipolytica*-based consolidated bioprocess concept and reveal further ways to improve performance. In particular, the results obtained with swollenin provide insight into how this type of protein could be used in an advanced biorefinery concept involving a swollenin-mediated biomass pretreatment step and a CBP unit operation. Further strain engineering combined with appropriate process design will undoubtedly lead to much better performances in the future.

## Methods

### Strains and media

The genotypes of the microbial strains used in the present study are summarized in Table 1. *E. coli* DH5 was purchased from Invitrogen (Paisley, UK) and used for plasmid construction. The *Y. lipolytica* strains were routinely cultivated in a medium composed of 1% w/v yeast extract, 1% w/v Bacto peptone, and 1% w/v glucose (YPD), solid media contained 1.5% agar. Transformants were selected on solid YNB medium (0.17% w/v YNB, 1% glucose or cellobiose w/v, 0.5% w/v ammonium chloride, with (for Ura^+^) or without (for Leu^+^) 0.2% w/v casamino acids and 50 mM sodium–potassium phosphate buffer, pH 6.8), supplemented with uracil (440 mg/L) or leucine (440 mg/L) depending on the auxotrophic requirements. The detection of xylanase activity in solid YNB medium was achieved by incorporating 2.0% w/v AZCL-arabinoxylan (Megazyme). For cellulase characterization, enzymes were produced in YTD medium (1% w/v yeast extract, 2% w/v tryptone, 5% w/v glucose and 100 mM phosphate buffer, pH 6.8). To evaluate the growth of engineered cellulolytic *Y. lipolytica* on cellulose, transformants were aerobically cultivated in defined medium containing vitamins, trace elements [50], and salts, including 3.5 g/L (NH_4_)_2_SO_4_, 3.0 g/L K_2_HPO_4_, 3.0 g/L NaH_2_PO_4_, and 1.0 g/L MgSO_4_·7H_2_O with 27.5 g/L CIMV-cellulose (91% w/v cellulose, provided by CIMV S.A.) [44], or 25 g/L Avicel PH-101 (Sigma) or 46 g/L wheat straw (particle size ~ 0.5 mm, 27% w/w arabinoxylan, and 44% w/w cellulose) [51]. In addition, ascorbate (1 mM) was added into the cultures as the reducing agent to reactivate LPMOA.

### Plasmid constructions

The plasmids constructed in the present study are summarized in Table 3 and all primers are listed in Table 4. Briefly, the total RNA from 5-day cultured *T. reesei* QM9414 was isolated using RNeasy Plus Mini Kit (QIAGEN) and reverse transcription was performed with iScript™ cDNA Synthesis Kit (BIO-RAD) according to the manufacturer’s instructions. *LPMOA* gene (formerly known as GH61A, GenBank accession code: Y11113.1) was amplified from the cDNA of *T. reesei* by PCR using F (1) as forward primer and R (1) as reverse primer. A 15-base pair homologous sequence of the target plasmid was introduced into the upstream and downstream of each gene during PCR amplification. After that, the gene encoding LPMOA was fused with the PCR fragment (primers JMP1F/JMP1R) of secretion vector JMP62UraTEF under the control of TEF promoter and the pre-pro sequence of lipase 2 of *Y. lipolytica* by In-Fusion Cloning (Clontech). *XYN II* gene (GenBank accession code: XM_006968885.1), encoding *T. reesei* xylanase II, was amplified from the plasmid JMP63UraXYNII (kindly provided by Dr. Cédric Montanier, INSA-Toulouse) by PCR using primers F (2) and R (2) and fused with secretion vector JMP62UraTEF as described above. The *SWO1* gene (GenBank accession code: AJ245918.1), encoding *T. reesei* swollenin, was synthesized by GenScript (USA) after codon optimization based on the codon bias of *Y. lipolytica* (Additional file 2), and cloned into the plasmid JMP62UraTEF.

**Table 3:** Plasmids used or created in the present study

Plasmids	Description	Source of reference
JMP62UraTEF	*URA3*, *pTEF*	[52]
JMP62LeuTEF	*LEU2*, *pTEF*	[52]
JMP62UraTB1his	*URA3*, *pTEF*-*BGL1*-*His6*	[57]
PUB4-CRE	*hph*, *hp4d*-*CRE*	[53]
JMP62UraHTEF	*URA3*, *pHTEF*	[30]
JMP62LeuHTEF	*LEU2*, *pHTEF*	[30]
JMP62hphTEF	*Hph*, *pTEF*	This investigation
JMP62LeuHTEF-EG1	*LEU2*, *pHTEF*-*EG I*	This investigation
JMP62UraXYNIIhis	*URA3*, *pTEF*-*XYNII*-*His6*	This investigation
JMP62UraLPMOAhis	*URA3*, *pTEF*-*LPMOA*-*His6*	This investigation
JMP62UraSWO1his	*URA3*, *pTEF*-*SWO1*-*His6*	This investigation
JMP62UraXYNII	*URA3*, *pTEF*-*XYNII*	This investigation
JMP62LeuXYNII	*LEU2*, *pTEF*-*XYNII*	This investigation
JMP62hphXYNII	*hph*, *pTEF*-*XYNII*	This investigation
JMP62UraLPMOA	*URA3*, *pTEF*-*LPMOA*	This investigation
JMP62LeuLPMOA	*LEU2*, *pTEF*-*LPMOA*	This investigation
JMP62hphLPMOA	*hph*, *pTEF*-*LPMOA*	This investigation
JMP62hphLPMOA/XYNII	*hph*, *pTEF*-*LPMOA*, *pTEF*-*XYNII*	This investigation
JMP62UraSWO1	*LEU2*, *pTEF*-*SWO1*	This investigation
JMP62Leu SWO1	*LEU2*, *pTEF*-*SWO1*	This investigation
JMP62hph SWO1	*hph*, *pTEF*-*SWO1*	This investigation

**Table 4:** Sequences of the oligonucleotide primers used in this study

Primer names	Sequence (5′–3′), 15-bp homologous sequence for infusion is underlined
F1	GTTCTCCAGAAGCGACATGGACATATTAATGACATTGTCATCAACG
R1	CACAGACACCCTAGGCTAGTTAAGGCACTGGGCGTAGTAGGG
F2	GTTCTCCAGAAGCGACAGACCATCCAGCCCGGCACC
R2	CACAGACACCCTAGGTTAGCTCACGGTGATAGAGGCAGAGCCA
JMP1F	CCTAGGGTGTCTGTGGTATCTAAGCTATT
JMP1R	TCGCTTCTGGAGAACTGCGG
F3	ACACCCGAAGGATCCCACAATGAAGCTTTCCACCATCC
R3	ATGGTGATGATGGTGGCTCACGGTGATAGAGGCAGAG
R4	ATGGTGATGATGGTGGTTAAGGCACTGGGCGTAGTAGGG
R5	ATGGTGATGATGGTGGTTCTGGGAAAACTGGACGCC
JMP2F	CACCATCATCACCATCATTAAAACT
JMP2R	GGATCCTTCGGGTGTGAGTTG
HphF	CCACACACATCCACAATGAAAAAGCCTGAACTCACCGCGA
HphR	TAGCAGGGCAGGGCCCTATTCCTTTGCCCTCGGACGAGTG
LHFF	GGCCCTGCCCTGCTAATGAAATG
LHFR	TGTGGATGTGTGTGGTTGTATGTGTGATG
XLF	GC*TCTAGA*CGATGCCGCCGCAAGGAATG (*Xba*I)
XLR	CG*TCTAGA*TGGAATTCGATTTGTCTTAGAGGAACGCA (*Xba*I)

For the expression of His-tagged proteins, *XYNII*, *LPMOA*, and *SWO1* were cloned by PCR with F3 as forward primer and R (3–5) as reverse primers using the expression vectors constructed in last step as template, and fused with PCR fragment (primers JMP2F/JMP2R) of the vector JMP62UraTB1his [30]. This enables the addition of a sequence encoding 6-histidine at the C-terminus of the proteins.

To improve the expression level of EG I, the plasmid JMP62UraEG1 was digested using *Bam*HI/*Avr*II, and the *EG* I gene was recovered and then inserted into the corresponding sites of the previously constructed plasmid JMP62UraHTEF under the control of TEF promoter with an enhancer comprising 4 tandem copies of upstream activation sequences (4UASs) [30].

After construction, all expression vectors were verified by DNA sequencing (GATC Biotech, Konstanz, Germany). For *Y. lipolytica* transformation, vectors were digested using *Not*I, thus generating a linear DNA with Zeta sequences at both extremities. Then the gel purified expression cassettes were introduced into the Zeta docking platform of *Y. lipolytica* JMY1212 Zeta for the expression of single recombinant protein, or randomly into the genome of ∆*pox*B12 strain, for co-expression of multiple cellulases, using the lithium acetate method [52]. For the latter case, the *LoxP*-Cre recombination system was used for marker rescue and to ensure the multistep insertion of the target genes [53]. However, after the integration of six cellulase genes (*BGL*1, *BGL*2, *EGI*, *EGII*, *CBHI*, and *CBHII*), the removal of the selection markers (*URA*3 and *LEU*2) from cellulolytic *Y. lipolytica* caused loss of the previously introduced genes, probably due to the presence of multiple *LoxP* sites. In order to further introduce accessory proteins into cellulolytic *Y. lipolytica*, the hygromycin phosphotransferase coding gene hph, conferring hygromycin resistance to *Y. lipolytica*, was amplified from the vector pUB4-Cre by PCR using primers HphF and HphR. Then, the hph gene was fused with DNA fragment amplified from the vectors JMP62LeuTEFLPMOA, JMP62LeuTEFXYNII, and JMP62LeuTEFSWO1 using primers LHFF and LHFR, respectively. The plasmid for co-expression of *Tr*LPMOA *and Tr*XYNII was constructed by insertion of the expressing cassette containing *TrLPMOA* under the control of TEF promoter, obtained by PCR amplification from the vector JMP62UraTEFLPMOA using primers XLF and XLR, into the *Xba*I site of the vector JMP62hphTEFXYNII. The successful multiple integration of the heterologous genes into the genome of *Y. lipolytica* was verified by PCR using gene-specific primers (Additional file 1: Table S1). In addition, transformants expressing multiple enzymes were tested for growth on cellobiose, and for degradation of Azo-CM-Cellulose and AZCL-arabinoxylan. Clones displaying both activities were retained for further analysis. Table 3 summarizes the expressed cellulase genes and their corresponding *Y. lipolytica* transformants.

### Protein production and hydrolytic activity assay

Recombinant protein production by *Y. lipolytica* was carried out in liquid YTD medium in shake flask at 28 °C and 120 rpm for 5 days. PASC was prepared from Avicel PH-101 as previously described [54]. The overall cellulolytic activities were measured on CMC, PASC, CIMV-cellulose, and Avicel PH-101 using a previously described method with slight modifications [55]. Briefly, the reaction mixture contained 1% (w/v) cellulosic substrate, 50 mM citrate buffer (pH 4.8), and proper volume of diluted enzyme solution.

Xylanase activity was measured on 2% (w/v) beechwood xylan (Megazyme) in 50 mM citrate buffer (pH 6.0). The reaction was conducted at 50 °C for 10 min.

The reducing sugars were quantified using the dinitrosalicylic acid (DNS) reagent [30]. One unit of activity (U) was defined as the amount of enzyme or culture supernatant required to release 1 μmol of reducing sugars per min for xylanase activity, or 1 mg of reducing sugars per min for cellulolytic activity.

In addition, glucose and xylose were measured using an Aminex HPX87-H column (Bio-Rad Laboratories, Germany), operating at 50 °C using a mobile phase (5 mM H_2_SO_4_) flowing at a rate of 0.5 mL/min. Glucose and xylose were detected using a Shodex RI-101 refractive index detector (Showa Denko, New York, NY, USA).

All protein concentrations were measured using the Bradford method and bovine serum albumin as a standard [56]. All enzymatic activity measurements were performed in triplicate unless otherwise stated.

### SDS-PAGE and Western blot analysis

SDS-PAGE was conducted using Mini-PROTEAN TGX Stain-Free precast gels (Biorad) according to the manufacturer’s instructions. Fifteen microlitre of culture supernatant or enzyme solution was loaded into each well. Western blotting of proteins was performed as described previously [57]. Crude supernatant of *Y. lipolytica* JMY1212 expressing LPMOA and SWO1 fused with the His6 tag were concentrated tenfold by ultrafiltration on an Omega™ membrane disc filter with a 10 kDa cut off (Pall, France). Blots were sequentially treated with mouse non-position-specific His-Tag antibody 1:2500 (THE™ from Genscript, Piscataway, NJ, US) and the alkaline phosphatase-conjugated goat anti-mouse IgG. Phosphatase activity was detected by NBT/BCIP as substrate.

### Purification and deglycosylation of recombinant proteins

*Yarrowia lipolytica* JMY1212 expressing His6-tagged XYNII, LPMOA and SWO1, respectively, was grown in 200 mL YTD medium at 120 rpm, 28 °C for 48 h. After centrifugation (8000×*g* for 5 min at 4 °C), the supernatant was concentrated tenfold by ultrafiltration with an Omega™ membrane disc filter at 10 kDa cut off (Pall, France), and applied to 2 mL of TALON Metal Affinity Resin (Clontech, Takara-Bio, Kyoto, Japan). Subsequently, protein was eluted using buffer containing imidazole according to the manufacturer’s instructions. Deglycosylation was carried out by treating the purified proteins with endoglycosidase H (New England Biolabs, Beverly, MA, USA) to remove N-linked carbohydrates at 37 °C for 1 h. Protein samples were analyzed by SDS-PAGE and visualized with colloidal Coomassie blue staining.

### Amplex Red/horseradish peroxidase assay

The oxygen reactivity of *Tr*LPMOA was measured in a quantitative time resolved assay of H_2_O_2_ using Amplex Red and horseradish peroxidase as described previously [8, 22]. The reaction mixture (100 μL) contained 100 mM sodium acetate buffer (pH 6.0), 50 μM Amplex Red reagent (Invitrogen, France), 7.14 U/mL horseradish peroxidase, 0.1–0.5 μM LPMOA and 30 μM ascorbate as reductant, and was assembled in the well of a 96-well microtiter plate and incubated at 30 °C. The fluorescence (excitation and emission wavelengths of 560 and 595 nm, respectively) was measured using a Tecan Infinite M200Pro plate reader (Tecan, Männedorf, Switzerland). The activity of *Tr*LPMOA was derived from the data using a standard H_2_O_2_ calibration curve. The inhibition of H_2_O_2_ production was used to test the activity of *Tr*LPMOA on various β-glucan and xylan substrates as described previously [41]. A final concentration of 1.0% (w/v) PASC, CMC, Avicel PH-101, barley glucan (β-1,3; 1,4), and xylan was added into the above reaction mixture. All measurements were performed in triplicates.

### Cello-oligosaccharide cleavage assay

The cleavage of cello-oligosaccharides by LPMOA was assayed by incubating 16 mg of *Tr*LPMOA, 1 mM of ascorbate, and 5 mM cello-oligosaccharides (DP4-DP6) in 50 mM sodium acetate buffer (pH 4.8) at 50 °C for 24 h under shaking (1000 rpm). To stop the reaction, samples were boiled (100 °C for 10 min) and then cooled before adding an aliquot of Novozyme 188 β-glucosidase (gift from Novozyme, Denmark) (12.0 IU/g cello-oligosaccharides, 810 IU/mL). After that, the reaction was incubated for a further 12 h and then, the oxidized mono-saccharides in the supernatant were measured using a d-gluconic acid/d-glucono-δ-Lactone assay kit (Megazyme) according to manufacturer’s instructions. All assays were carried out in triplicate.

### Study of the synergy of LPMOA and SWO1 on enzymatic hydrolysis of Avicel

Enzymatic hydrolysis of cellulose was carried out in 50 mM citrate buffer (pH 4.8) containing 2.0% (w/v) microcrystalline cellulose (Avicel PH-101, Sigma). Celluclast 1.5 L (60 FPU/mL) and β-glucosidase (810 IU/mL, Novozyme 188) were added at 5.0 FPU/g-cellulose and 12.0 IU/g-cellulose, respectively. The reaction (1 mL) was conducted in Eppendorf tubes (2 mL) using a thermomixer at 50 °C and 1000 rpm. To study the synergistic effect of *Tr*LPMOA on the enzymatic hydrolysis of cellulose, different amounts of this protein (5, 10, and 15 mg-protein/g-cellulose, respectively) were added to the reaction mixtures containing Avicel (2.0% w/v). In addition, ascorbate (1 mM) was added into the reactions as the reducing agent to reactivate LPMOA. To study the synergistic effect of swollenin on the enzymatic hydrolysis of cellulose, this protein was either added into reaction mixture together with cellulases at different concentrations (5, 10, and 15 mg-swollenin/g-cellulose respectively) or, incubated with the Avicel at 50 °C under shaking for 24 h prior to the addition of cellulases. Control experiments were conducted under the same conditions, with or without, BSA (at 5, 10, and 15 mg-protein/g-cellulose). Samples were taken at regular intervals to determine the concentration of reducing sugars using DNS.

### Enzymatic hydrolysis of cellulosic feedstocks

Enzymatic hydrolysis of different cellulosic feedstocks was carried out in 50 mM citrate buffer (pH 4.8). CIMV-cellulose, Avicel and wheat straw, respectively, was added into the reaction to yield a final cellulose concentration of 2.0% (w/v). Cellic^®^ CTec2 (a gift from Novozyme) or concentrated secretome of *Y. lipolytica* YLC11 was added at 10.0 FPU/g-cellulose. The reaction mixture was incubated at 50 °C under shaking for 120 h. Samples were taken at regular intervals to determine the concentration of glucose using HPLC.

### Growth of yeast expressing multiple cellulases on cellulosic feedstocks

Yeast growth on different cellulosic feedstock was performed in 50 mL defined medium containing CIMV-cellulose, Avicel cellulose or wheat straw in 250-mL Erlenmeyer flasks. Yeasts were pre-cultivated in defined media on glucose until mid-exponential phase and the cells were collected by centrifugation. After washing with deionized water, the cells were used to inoculate the defined medium containing cellulose to yield an initial biomass concentration of 1.0 g-DCW/L. In addition, CIMV-cellulose, Avicel cellulose, and wheat straw were incubated with swollenin at 50 °C under shaking for 24 h prior to the inoculation of strain YLC11. All the cultivations were conducted at 28 °C and under shaking at 120 rpm. Samples were taken at the end of 5 days to determine concentrations of biomass and residual cellulose (see below). The data are presented as mean values of at least three biological replicates ± standard deviation.

### Analysis of cellulose residues and determination of dry cell weight

The quantification of residual cellulose and dry cell matter was conducted as previously described with slight modifications [30]. Briefly, the cell pellets mixed with cellulose from the above cultures were harvested by centrifugation at 8000×*g* for 10 min at 4 °C. After two washes with distilled water, the collected cellulose-cell pellet was freeze-dried and weighed. The amount of remaining cellulose and hemicellulose was calculated from the total glucose and xylose released from diluted acid hydrolysis of the residues with 2.5% sulfuric acid at 121 °C for 1 h. Dry cell weight was deduced by subtracting the amount of cellulose or lignocellulose from the weight of cellulose or lignocellulose-cell pellet. Glucose and xylose were measured by HPLC as described before [57]. The biomass yield was calculated as the ratio of the amount of biomass obtained divided by the amount of carbon source consumed.

## Additional files

**Additional file 1: Figure S1.** PCR verification of *Y. lipolytica* transformants expressing multiple cellulases and accessory proteins (A) YLC7, Lane 1 to 6: *BGL1, BGL2, 4UASTrEGI, TrEGII, 4UASNcCBHI, 4UASTrCBHII*; (B) YLC8, Lane 1 to 6: *BGL1, BGL2, 4UASTrEGI, TrEGII, 4UASNcCBHI, 4UASTrCBHII, TrXYNII*; (C) YLC9, Lane 1 to 7: *BGL1, BGL2, 4UASTrEGI, TrEGII, 4UASNcCBHI, 4UASTrCBHII*, *TrLPMOA*; (D) YLC10, Lane 1 to 7: *BGL1, BGL2, 4UASTrEGI, TrEGII, 4UASNcCBHI, 4UASTrCBHII*, *TrSWO1*; (D) YLC11, Lane 1 to 8: *BGL1, BGL2, 4UASTrEGI, TrEGII, 4UASNcCBHI, 4UASTrCBHII*, *TrXYNII*, *TrLPMOA*. **Figure S2.** Western blot analysis of the heterologous rh*Tr*EGI protein secreted by the engineered *Y. lipolytica* strains: lane 1, Endo H-treated secretome of YLC6b (20 μL); lane 2, Endo H-treated secretome of YLC7b (20 μL). **Figure S3.** Characterization of the recombinant XYNII expressed in *Y. lipolytica*. (a) Effect of pH on the activity of rhXYNII; (B) Effect of temperature on the activity of rhXYNII. **Figure S4.** Screening of *Y. lipolytica* expressing cellulases and accessory enzymes on YNB indication plate containing supplemented with 0.2% w/vAzo-CM-Cellulose. Lane 1, *Y. lipolytica* control; Lane 2 to 4, YLC8, YLC9 and YLC10. **Figure S5.** The growth of *Y. lipolytica* in defined medium containing 10 g/L gluconic acid or 10 g/L glucose. **Table S1.** The sequences of the oligonucleotide primers used for PCR verification of *Y. lipolytica*-transformants. **Table S2.** Comparison of cellulose utilization and biomass yield of cellulolytic *Y. lipolytica* grown on different cellulosic substrates for 120 h in aerobic cultivation without the addition of ascorbic acid.

**Additional file 2.** Nucleotide sequence of codon-optimized SWO1.

## Abbreviations

BCIP: 5-bromo-4-chloro-3-indolyl phosphate
BGL: β-glucosidase
CBH: cellobiohydrolase
CBP: consolidated bioprocessing
CMC: carboxymethyl cellulose
DCW: dry cell weight
EG: endoglucanase
Endo H: endoglycosidase
LCB: lignocellulosic biomass
LPMO: lytic polysaccharide monooxygenase
Mw: molecular weight
NBT: nitroblue tetrazolium
*Nc*: *Neurospora crassa*
PAGE: polyacrylamide gel electrophoresis
PASC: phosphoric acid swollen cellulose
r: recombinant proteins
rh: His6-tagged recombinant proteins
HPAEC: high performance anion exchange chromatography
SWO: swollenin
*Tr*: *Trichoderma reesei*
*yl*: *Y. lipolytica*
