# Supplementary material for: Expressing accessory proteins in cellulolytic Yarrowia lipolytica to improve the conversion yield of recalcitrant cellulose
Source: Biotechnol Biofuels. 2017 Dec 11;10:298. doi: 10.1186/s13068-017-0990-y (PMC5724336; doi:10.1186/s13068-017-0990-y)
Supplement: Supplementary file 1 — Additional file 1: Figure S1. PCR verification of Y. lipolytica transformants expressing multiple cellulases and accessory proteins (A) YLC7, Lane 1 to 6: BGL1, BGL2, 4UASTrEGI, TrEGII, 4UASNcCBHI, 4UASTrCBHII; (B) YLC8, Lane 1 to 6: BGL1, BGL2, 4UASTrEGI, TrEGII, 4UASNcCBHI, 4UASTrCBHII, TrXYNII; (C) YLC9, Lane 1 to 7: BGL1, BGL2, 4UASTrEGI, TrEGII, 4UASNcCBHI, 4UASTrCBHII, TrLPMOA; (D) YLC10, Lane 1 to 7: BGL1, BGL2, 4UASTrEGI, TrEGII, 4UASNcCBHI, 4UASTrCBHII, TrSWO1; (D) YLC11, Lane 1 to 8: BGL1, BGL2, 4UASTrEGI, TrEGII, 4UASNcCBHI, 4UASTrCBHII, TrXYNII, TrLPMOA. Figure S2. Western blot analysis of the heterologous rhTrEGI protein secreted by the engineered Y. lipolytica strains: lane 1, Endo H-treated secretome of YLC6b (20 μL); lane 2, Endo H-treated secretome of YLC7b (20 μL). Figure S3. Characterization of the recombinant XYNII expressed in Y. lipolytica. (a) Effect of pH on the activity of rhXYNII; (B) Effect of temperature on the activity of rhXYNII. Figure S4. Screening of Y. lipolytica expressing cellulases and accessory enzymes on YNB indication plate containing supplemented with 0.2% w/vAzo-CM-Cellulose. Lane 1, Y. lipolytica control; Lane 2 to 4, YLC8, YLC9 and YLC10. Figure S5. The growth of Y. lipolytica in defined medium containing 10 g/L gluconic acid or 10 g/L glucose. Table S1. The sequences of the oligonucleotide primers used for PCR verification of Y. lipolytica-transformants. Table S2. Comparison of cellulose utilization and biomass yield of cellulolytic Y. lipolytica grown on different cellulosic substrates for 120 h in aerobic cultivation without the addition of ascorbic acid. [file 13068_2017_990_MOESM1_ESM.docx]

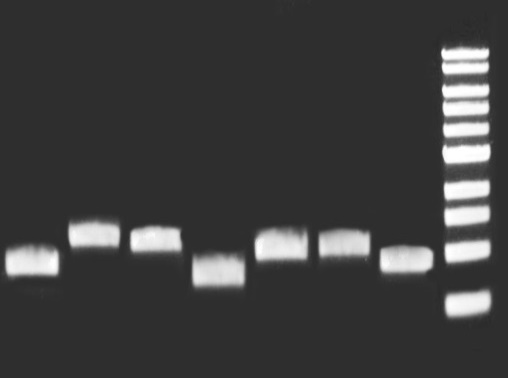

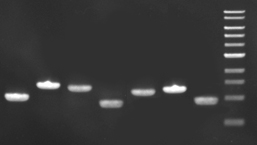

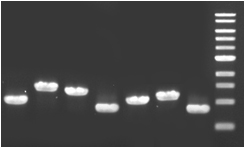

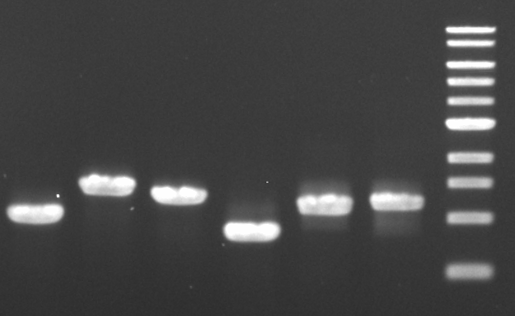


**B** 1 2 3 4 5 6 7

**A** 1 2 3 4 5 6

**C** 1 2 3 4 5 6 7

**D** 1 2 3 4 5 6 7


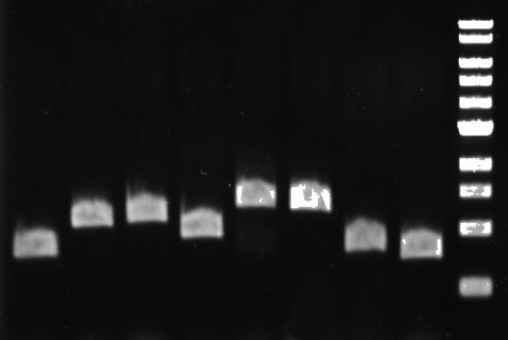


**E** 1 2 3 4 5 6 7 8

Figure S1 PCR verification of *Y. lipolytica* transformants expressing multiple cellulases and accessory enzymes (A) YLC7, Lane 1 to 6: *BGL1, BGL2, 4UASTrEGI, TrEGII, 4UASNcCBHI, 4UASTrCBHII*; (B) YLC8, Lane 1 to 6: *BGL1, BGL2, 4UASTrEGI, TrEGII, 4UASNcCBHI, 4UASTrCBHII, TrXYNII*; (C) YLC9, Lane 1 to 7: *BGL1, BGL2, 4UASTrEGI, TrEGII, 4UASNcCBHI, 4UASTrCBHII*, *TrLPMOA*; (D) YLC10, Lane 1 to 7: *BGL1, BGL2, 4UASTrEGI, TrEGII, 4UASNcCBHI, 4UASTrCBHII*, *TrSWO1*; (D) YLC11, Lane 1 to 8: *BGL1, BGL2, 4UASTrEGI, TrEGII, 4UASNcCBHI, 4UASTrCBHII*, *TrXYNII*, *TrLPMOA*.


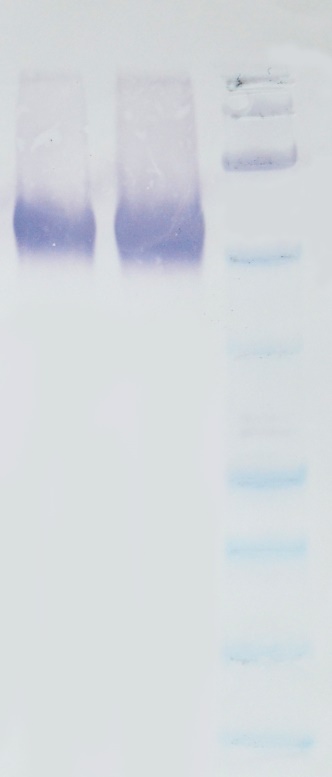


**1 2**

150kD

100

75

50

37

Figure S2 Western blot analysis of the heterologous rh*Tr*EGI protein secreted by the engineered *Y. lipolytica* strains: lane 1, Endo H-treated secretome of YLC6 (20 μL); lane 2, Endo H-treated secretome of YLC7 (20 μL).

Figure S3 Characterization of the recombinant XYNII expressed in *Y. lipolytica*. (a) Effect of pH on the activity of rhXYNII; (b) Effect of temperature on the activity of rhXYNII.

1 2 3 4


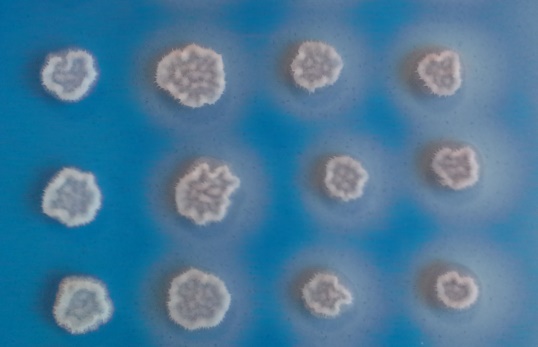


Figure S4 Screening of *Y. lipolytica* expressing cellulases and accessory enzymes on YNB indication plate containing supplemented with 0.2% w/v Azo-CM-Cellulose. Lane 1, *Y. lipolytica* control; Lane 2 to 4, YLC8, YLC9 and YLC10.


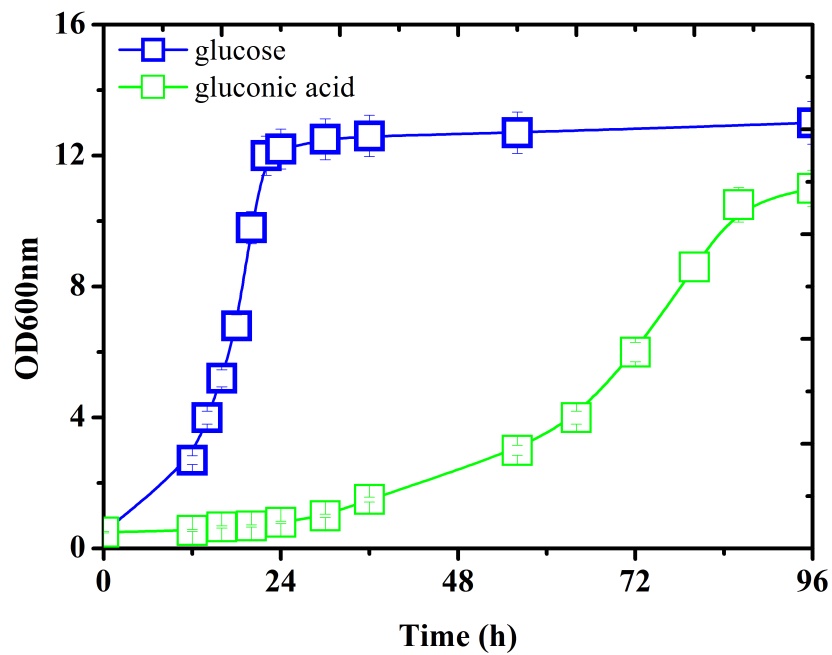


Figure S5 The growth of *Y. lipolytica* in defined medium containing 10 g/L gluconic acid or 10 g/L glucose.

Table S1 The sequences of the oligonucleotide primers used for PCR verification of *Y. lipolytica*-transformants

| Primer names | Sequence (5’-3’) |
| --- | --- |
| VBGL1A | TTGACCCAGTAGCGGACCCAA |
| VBGL1B | GCCGACATTAGCCCTAACAGCAT |
| VBGL2A | GGTTGGCGGCGCATTTGT |
| VBGL2B | TGTCGTCCACTCGGCTTTCATC |
| V4UASEGIA | CACTTGCCGTTAAGGGCGTAGGGT |
| V4UASEGIB | CTGGCTGTTGTCGTTCCAAATGCTG |
| VEGIIA | CTTGCCGTTAAGGGCGTAGGG |
| VEGIIB | CCATCGAGCATAATTGTGGATGTCG |
| VCBHIA | TTTGCTTTGTGGTTGGGACTTTAGC |
| VCBHIB | GGTTCGAGATCCGATGTTAGTGGAGTA |
| VCBHIIA | TTTGCTTTGTGGTTGGGACTTTAGCC |
| VCBHIIB | AGGCGTTGGACCAGCCGTGA |
| V4UASCBHIA | CGCCGCAAGGAATGGTGCA |
| V4UASCBHIB | GGTTCGAGATCCGATGTTAGTGGAGTA |
| V4UASCBHIIA | CGCCGCAAGGAATGGTGCA |
| V4UASCBHIIB | CCGATCCGACTGGAGGTACTCTGGTAG |
| VXYNA | CCCAAATTGACCCAGTAGCGGACCCA |
| VXYNB | GAGAAGTAGCCCTCGACGGCGACGATC |
| VLPMOA | TTGACCCAGTAGCGGACCCAACCC |
| VLPMOB | TGATCTCGTGGCGGAGCACGTAATT |
| VSWOA | TTTTGCTTTGTGGTTGGGACTTTAGCC |
| VSWOB | GGGTGTAGTAGTCGCCGTTGGGAGC |

Table S2 Comparison of cellulose utilization and biomass yield of cellulolytic *Y. lipolytica* grown on different cellulosic substrates for 120h in aerobic cultivation without the addition of ascorbic acid^a^

| Strains | CIMV-cellulose consumed % | Biomass yield % | Cellulose consumed % (wheat straw) | Biomass yield % |
| --- | --- | --- | --- | --- |
| YLC9 | 64.9 ± 0.5 | 0.37 ± 0.03 | 16.0 ± 0.6 | 0.16 ± 0.01 |
| YLC11 | 67.2 ± 0.8 | 0.38 ± 0.03 | 17.8 ± 0.5 | 0.15 ± 0.02 |
| ^b^YLC11+ S | 68.5 ± 1.0 | 0.38 ± 0.02 | 20.9 ± 0.7 | 0.16 ± 0.01 |

^a^The results were calculated from at least three biological replicates, and are given as the mean value ± standard deviation. The initial cellulose content was 25 g/L for all the substrates.

^b^The cellulose was treated by SWO1 at the dosage of 15 mg/g cellulose for 24 h before enzymatic hydrolysis.
