# Supplementary material for: Expressing accessory proteins in cellulolytic Yarrowia lipolytica to improve the conversion yield of recalcitrant cellulose
Source: Biotechnol Biofuels. 2017 Dec 11;10:298. doi: 10.1186/s13068-017-0990-y (PMC5724336; doi:10.1186/s13068-017-0990-y)
Supplement: Supplementary file 2 — Additional file 2. Nucleotide sequence of codon-optimized SWO1. [file 13068_2017_990_MOESM2_ESM.pdf]

Nucleotide sequence of codon-optimized SWO1

Note that this nucleotide sequence was codon-optimized based on the codon bias of *Y. lipolytica* and is started with prepro sequence of Lip2 of *Y. lipolytica* (underlined)

>*TrSWO1*
ATGAAGCTTTCCACCATCCTTTTCACAGCCTGCGCTACCCTGGCTGCCGCCCTCCCTTCCCCCATCACTC
CTTCTGAGGCCGCAGTTCTCCAGAAGCGAGCCCAGCAGAACTGTGCCGCTCTCTTTGGACAGTGTGGC
GGAATCGGTTGGTCGGGTACTACCTGTTGCGTTGCCGGTGCCCAGTGCTCCTTCGTGAACGACTGGTAC
TCGCAGTGTCTCGCTTCCACCGGAGGTAACCCTCCTAACGGTACCACTTCCTCTTCGCTGGTGTCCCGA
ACCTCCTCTGCTTCGTCCTCTGTTGGCTCGTCCTCTCCCGGAGGCAACTCTCCTACTGGTTCTGCCTCG
ACCTACACCACTACCGACACTGCCACCGTTGCTCCCCACTCCCAGTCTCCCTACCCTTCCATCGCCGCT
TCGTCCTGTGGATCTTGGACCCTCGTCGACAACGTGTGCTGTCCTTCTTACTGCGCTAACGACGATACC
TCGGAGTCCTGCTCTGGCTGTGGTACCTGCACTACCCCCCCTTCTGCCGACTGTAAGTCGGGTACCATG
TACCCCGAGGTTCACCATGTCTCTTCGAACGAGTCCTGGCACTACTCGCGATCCACTCATTTCGGACTG
ACCTCTGGTGGAGCTTGTGGATTTGGCCTGTACGGACTCTGCACCAAGGGCTCCGTCACTGCCTCTTG
GACCGACCCCATGCTCGGAGCCACTTGTGATGCTTTCTGCACCGCCTACCCCCTGCTCTGCAAGGACCC
TACCGGTACTACCCTGCGAGGAAACTTCGCCGCTCCCAACGGCGACTACTACACCCAGTTTTGGTCCTC
TCTGCCTGGTGCCCTCGATAACTACCTGTCCTGTGGAGAGTGCATCGAGCTCATTCAGACTAAGCCCGA
CGGTACCGATTACGCTGTTGGCGAGGCCGGTTACACTGACCCCATCACCCTGGAGATTGTCGATTCTTG
CCCTTGTTCTGCTAACTCGAAGTGGTGCTGTGGTCCTGGAGCTGACCACTGTGGAGAGATTGATTTCAA
GTACGGTTGCCCCCTGCCTGCTGACTCCATCCATCTGGACCTCTCTGATATTGCCATGGGACGACTCCA
GGGCAACGGTTCCCTGACTAACGGCGTCATCCCCACCCGATACCGACGAGTCCAGTGCCCTAAGGTCG
GCAACGCTTACATTTGGCTCCGAAACGGCGGTGGACCCTACTACTTTGCCCTGACTGCTGTTAACACCA
ACGGACCTGGCTCTGTCACCAAGATCGAGATTAAGGGCGCTGACACCGATAACTGGGTGGCCCTGGTT
CACGACCCCAACTACACCTCGTCCCGACCTCAGGAGCGATACGGTTCCTGGGTCATCCCCCAGGGTTC
TGGACCCTTCAACCTCCCTGTGGGAATTCGACTGACTTCCCCCACCGGCGAGCAGATCGTGAACGAGC
AGGCCATTAAGACTTTTACCCCTCCTGCCACCGGAGACCCCAACTTTTACTACATTGACATCGGCGTCC
AGTTTTCCCAGAACTAA
